# CLK2 blockade modulates alternative splicing compromising MYC‐driven breast tumors

**DOI:** 10.15252/emmm.201809213

**Published:** 2018-05-22

**Authors:** Fernando Salvador, Roger R Gomis

**Affiliations:** ^1^ Oncology Program Institute for Research in Biomedicine (IRB Barcelona) The Barcelona Institute of Science and Technology Barcelona Spain; ^2^ CIBERONC Barcelona Spain; ^3^ Universitat de Barcelona Barcelona Spain; ^4^ ICREA, Institució Catalana de Recerca i Estudis Avançats Barcelona Spain

**Keywords:** Cancer, Pharmacology & Drug Discovery

## Abstract

*MYC* oncogene overexpression/amplification is common in multiple human cancers, in which it regulates proliferation, apoptosis and cell metabolism, among other processes, and its expression associates with poor prognosis. Targeting MYC presents an exciting therapeutic possibility, but developing appropriate drugs that impair protein function remains challenging. Searching for alternative therapeutic options for treating aggressive MYC‐driven cancers is thus of high clinical interest. Intriguingly, MYC‐driven cancers present vulnerability against spliceosome inhibition. In this issue of *EMBO Molecular Medicine*, Iwai *et al* (2018) tackle targeting the splicing regulatory Cdc2‐like kinase (CLKs) family. They report that a novel, orally administered CLK2 inhibitor (T‐025) induces exon skipping, which results in cancer cell growth reduction, especially in breast cancer (BCa) MYC‐driven tumors.

Through alternative splicing (AS), a single mRNA precursor with diverse splice sites can produce distinct mRNAs, potentially generating multiple protein isoforms with diverse functional roles. Aberrant splicing is frequently observed in cancer cells, providing them with the possibility of creating aberrant proteins that confer a selective advantage to them. Specific mutations affecting the recognition of splicing sites in particular genes can generate aberrant isoforms with pro‐tumoural functions. Dysregulated expression or mutations in proteins related to the splicing machinery can contribute to generating a wide spectrum of AS isoforms that can affect the oncogenic process by favoring proliferation, angiogenesis, invasion, and/or drug resistance (Dvinge *et al*, 2016). Thus, pharmacological blockade of spliceosome function has been proposed as a potent strategy for treating cancer and other diseases (Bonnal *et al*, 2012).

The spliceosome machinery, comprising five small nuclear ribonucleoproteins (snRNPs) and a multitude of interacting proteins, catalyzes intron excision/exon ligation. AS is regulated by additional *trans*‐acting factors, such as serine/arginine‐rich proteins (SR), that binds to specific sequences within introns or exons and either facilitate or hinder snRNP recruitment. CLKs and SR protein kinases (SRPKs) phosphorylate SR proteins, regulating their subcellular localization (within nuclear speckles) and their splicing activities (Dvinge *et al*, 2016). Inhibition of the kinase activity of CLKs or SRPKs has been proposed as a therapeutic option in cancer (Bonnal *et al*, 2012). Several small molecules that inhibit these kinase activities have been designed, including TG003, an inhibitor of CLK1 and CLK4 (Muraki *et al*, 2004). However, finding CLK inhibitors that can be orally administered has proven to be extremely challenging, which has blocked their potential use in treating cancer.

In this work, Iwai *et al* (2018) designed a highly specific CLK2 inhibitor (T‐025), which is stable, orally available, and has anti‐tumorigenic properties *in vivo*. Initially, the authors developed and characterized a new class of CLK inhibitors derived from 7H‐pyrrolo[2,3‐*d*]pyrimidine (T‐025) that reduces phosphorylation of SR proteins and consequently impairs AS (mainly inducing exon skipping). *In vitro*, T‐025 decreased cell growth and increases apoptosis; *in vivo*, it showed anti‐tumorigenic effects upon treating mice harboring xenograft tumors from MDA‐MB‐468 breast cancer (BCa) cells. The authors performed complementary *in vivo* experiments using lung (NCI‐H1048), myeloid leukemia cancer cells (MV‐4‐11), and acute myeloid leukemia patient‐derived xenograft cell models, and elucidated doses that are well‐tolerated. At a molecular level, Iwai *et al* (2018) showed that T‐025 induces skipping of exon 7 of the ribosomal protein S6 kinase pre‐mRNA, *RPS6KB1* (*S6K*), and of exon 11 of the Bcl‐2‐associated transcription factor 1 pre‐mRNA, BCLAF1, among others (Fig 1). In the big picture, pathway analysis based on genes whose expression changed upon T‐025‐dependent exon skipping revealed that cell cycle, DNA repair, RNA transport, and apoptosis were substantially affected. Consistently, previous reports using earlier generations of CLK and SRPK inhibitors also showed that these modulate AS in genes such as *S6K*,* EGFR*,* EIF3D*, and *PARP*. These alternative mRNA variants are less stable, leading to decreased protein levels and cell growth suppression (Araki *et al*, 2015). Of note, the S6K protein is a downstream target of the AKT/m‐TOR pathway that promotes cancer cell survival (Sridharan & Basu, 2011).

**Figure 1:**
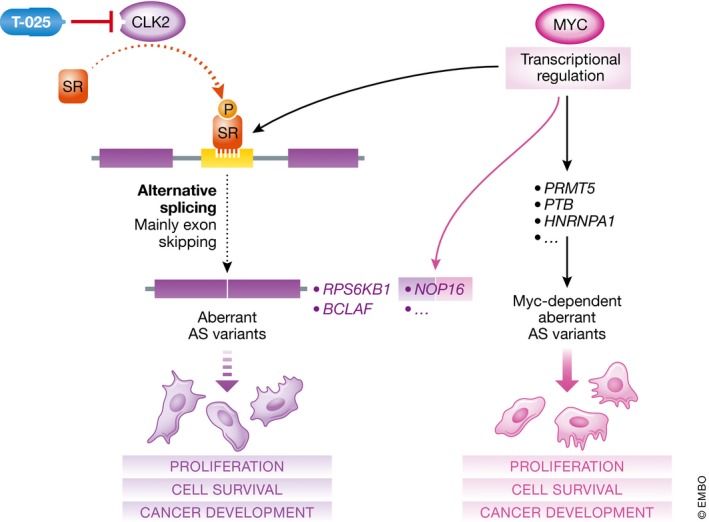
Overview of AS modulation by CLK2, MYC, and the T‐015 inhibitor CLK2 catalyzes the phosphorylation of SR proteins with a crucial role in exon recognition during AS. High expression of CLK2 drives the generation of aberrant AS variants, affecting *RPS6KB1*,* BCLAF*, and *NOP51* mRNAs (among others), which in turn are translated into oncogenic aberrant proteins. MYC transcriptionally regulates expression of several splicing activators or repressors (e.g., *PRMT5*,* PTB*, and *HNRNPA1*), contributing to generating CLK2‐independent aberrant AS variants and promoting malignant transformation. Transcriptional activity of MYC regulates the expression of *NOP16* and *SRF1*. Notably, *NOP16* mRNA is regulated by T‐025 at the pre‐mRNA level.

Patient stratification is currently a must to support the clinical developmental path. To identify biomarkers associated with T‐025 sensitivity, Iwai *et al* (2018) carried out an unbiased bioinformatics analysis using a panel of growth inhibition assays in 240 cancer cell lines and showed that T‐025 treatment reduced proliferation in both solid and hematological cancer cells. Interestingly, CLK2 expression was significantly associated with drug sensitivity. These data suggest that oncogenic activity is at least partially dependent on CLK2 expression, in agreement with previous reports suggesting that CLK2 acts as an oncogenic kinase in BCa (Yoshida *et al*, 2015). To corroborate these data, *in vitro* growth assays showed that cancer cell lines with high CLK2 protein levels displayed a striking T‐025‐dependent proliferation impairment as compared to those with low CLK2 expression. Furthermore, the degree of AS upon T‐025 treatment also correlated with the amount of CLK2 protein.

Strikingly, data from 169 cancer cell lines from the Cancer Cell Line Encyclopedia (CCLE) showed that only those lines harboring MYC amplification were significantly more sensitive to T‐025, making amplified MYC a strong biomarker candidate. To functionally validate these data, Iwai *et al* (2018) took advantage of an inducible system in human melanoma SK‐MEL28 cells that allows MYC overexpression to be controlled by doxycycline treatment. MYC expression converted SK‐MEL‐28 into T‐025‐sensitive cells, with decreased cell growth and more caspase‐3/7 activation. *In vivo* experiments with allograft tumors from a transgenic MMTV‐*MYC* BCa mouse model provided further support for the role of CLK inhibitors against MYC‐driven BCa tumors. Critically, data analyses showed that BCa patients who had both high CLK2 expression and *MYC* amplification—but not those who only had one or the other—had lower survival rates. After these findings have been functionally validated, the next questions to answer are whether these different aberrations are mechanistically connected, how they are related to AS, and what are the roles of these pathways during treatment response.

How do tumor cells uncouple T‐025 treatment from tumor growth? Which contextual determinants modulate T‐025 capacity to alter AS and growth and its dependency on MYC overexpression? Iwai *et al* (2018) analyzed the molecular connection between CLK2 and MYC in an attempt to address these questions. MYC is known to regulate mRNA splicing at different levels. For instance, MYC can transcriptionally regulate putative splicing regulators, such as PRMT5, an arginine methyltransferase important for the snRNP biogenesis. Further, MYC overexpression promotes intron retention and affects several essential cellular pathways. Therefore, inhibition of the spliceosome in MYC‐driven tumors has been suggested as an adequate pharmacological intervention in cancer (Hsu *et al*, 2015). To bypass the difficulties of inhibiting the MYC transcription factor, Iwai *et al* (2018) tested whether MYC activation regulates CLK2 expression. However, no changes were observed in either CLK2 mRNA or protein upon MYC overexpression. In addition, only five out of 546 AS events tested in several cancer cell lines were commonly regulated by T‐025 and MYC, suggesting that CLK2 and MYC regulated different splicing‐related genes. Interestingly, the authors found that pre‐mRNA splicing for *NOP16*, which encodes a multiprotein complex critical for ribosomal biogenesis that is associated with poor survival in BCa (Zhang *et al*, 2014), is regulated by T‐025. Moreover, previous reports show that MYC transcriptionally regulates the *NOP16* gene and *SRF1* (Butt *et al*, 2008), indicating that CLK2 may modulate MYC transcriptional targets (Fig 1). However, this molecular connection would only partially explain why MYC‐driven tumors are sensitive to T‐025. Identification of other genes/mRNA commonly regulated by CLK2 and MYC may help to understand the CLK inhibition vulnerability upon MYC induction. In fact, Iwai *et al* (2018) only establish a clear correlation between both MYC amplification and a high expression of CLK2 in BCa, suggesting that a significant heterogeneity exists among tumor types in terms of molecular mechanistics. Hematological cancers also seem to benefit from CLK2 inhibition, although CLK2 or MYC did not sensitize these cells to T‐025, thus indicating that alternative mechanisms account for such effects.

While Iwai *et al* (2018) showed that T‐025 also inhibited DYRK1A, a kinase that has been postulated as a tumor suppressor in some cancers (Liu *et al*, 2014), they point out that *DYRK1A* depletion in cancer cells blunt T‐025 treatment effects. Moreover, response sensitivity to T‐025 treatment did not correlate with *DYRK1A* expression levels, implying that T‐025 acts mainly through CLK2 inhibition.

Overall, this work has the potential to help improve cancer treatment. A clearer understanding of the underlying mechanism of action of T‐025 inhibitor as a function of the tumor type, as well as the interplay between MYC and CLK2, is now needed. Previously, an oral CLK1 inhibitor was shown to have therapeutic potential for Duchenne muscular dystrophy patients (Dvinge *et al*, 2016). By providing the first *in vivo* evidence of targeting CLKs for cancer treatment with orally available drugs, Iwai *et al* (2018) have contributed to establishing a new class of drugs acting on AS with potential clinical utility. Several antitumor drugs targeting the spliceosome have now been proposed; however, as splicing factors have been shown to act as oncogenic factors or tumor suppressors, elucidating the contextual determinants for each particular tumor type and case is a must. To this end, an in‐depth understanding of the role of CLK2 mRNA targets and their contextual determinants is necessary to eventually translate this compound into the clinic.
